# Molecular evolution in court: analysis of a large hepatitis C virus outbreak from an evolving source

**DOI:** 10.1186/1741-7007-11-76

**Published:** 2013-07-19

**Authors:** Fernando González-Candelas, María Alma Bracho, Borys Wróbel, Andrés Moya

**Affiliations:** 1 Joint Research Unit ‘Genómica y Salud’ CSISP (FISABIO), Instituto Cavanilles/Universidad de Valencia, c/ Catedrático José Beltrán, 2 46980-Paterna, Valencia, Spain; 2 Centro de Investigación Biomédica en Red en Epidemiología y Salud Pública (CIBERESP), Valencia, Spain; 3 Department of Genetics and Marine Biotechnology, Institute of Oceanology, Polish Academy of Sciences, Powstańców Warszawy 55, 81-712 Sopot, Poland; 4 Laboratory of Bioinformatics, Institute of Molecular Biology and Biotechnology, Adam Mickiewicz University in Poznań, Umultowska 89, 61-614 Poznań, Poland

**Keywords:** HCV, Outbreak, Forensics, Molecular epidemiology, Nosocomial transmission, Compartmentalization, Maximum likelihood, Dating transmission events, Viral evolution

## Abstract

**Background:**

Molecular phylogenetic analyses are used increasingly in the epidemiological investigation of outbreaks and transmission cases involving rapidly evolving RNA viruses. Here, we present the results of such an analysis that contributed to the conviction of an anesthetist as being responsible for the infection of 275 of his patients with hepatitis C virus.

**Results:**

We obtained sequences of the NS5B and E1-E2 regions in the viral genome for 322 patients suspected to have been infected by the doctor, and for 44 local, unrelated controls. The analysis of 4,184 cloned sequences of the E1-E2 region allowed us to exclude 47 patients from the outbreak. A subset of patients had known dates of infection. We used these data to calibrate a relaxed molecular clock and to determine a rough estimate of the time of infection for each patient. A similar analysis led to an estimate for the time of infection of the source. The date turned out to be 10 years before the detection of the outbreak. The number of patients infected was small at first, but it increased substantially in the months before the detection of the outbreak.

**Conclusions:**

We have developed a procedure to integrate molecular phylogenetic reconstructions of rapidly evolving viral populations into a forensic setting adequate for molecular epidemiological analysis of outbreaks and transmission events. We applied this procedure to a large outbreak of hepatitis C virus caused by a single source and the results obtained played a key role in the trial that led to the conviction of the suspected source.

## Background

Over the last few decades, molecular phylogenetic analyses of RNA viruses have been used frequently in the study of outbreaks and transmission chains [1-5]. Occasionally, these analyses have been used in courts to provide evidence in cases in which the ascertainment of the source of an outbreak would lead to economic compensation being paid to the infected victims [1,6]. On one occasion this type of evidence was accepted in a criminal case, and contributed to the conviction of a physician for the attempted homicide of his former lover by deliberate injection of blood infected with hepatitis C virus (HCV) and human immunodeficiency virus (HIV) [4]. These and other cases [7] usually involve only one or just a few transmission events produced in a short period of time.

HCV, a member of family *Flaviviridae*, is a positive sense single-stranded RNA virus. The size of its genome is about 9.6 kb. The genome encodes a polyprotein of about 3,000 amino acids in length, which is processed by host and viral proteases to release 3 structural (core, E1, E2) and 7 non-structural (p7, NS2-NS5B) proteins (reviewed in [8]). About 160 million people worldwide are infected with HCV [9] and around 80% of those infected progress to chronic infection. Up to 20% of infected individuals develop HCV-related complications, for example hepatocellular carcinoma, cirrhosis, or liver failure [10]. The natural history of the infection is quite variable, ranging from rapidly resolved acute infections to frequent cases of long periods of asymptomatic infection during which the virus can be transmitted to other hosts. The virus spreads primarily by blood-to-blood contact. The main routes of transmission are intravenous drug use, unscreened blood transfusions (in the developing world) and other incorrect medical procedures, especially those that involve reuse of needles and syringes [11].

In February 1998, a series of HCV infection cases were detected among patients who had undergone minor surgery at a private hospital in the city of Valencia, Spain. Public health officials launched an epidemiological investigation that revealed a likely common source for these new infections to be an anesthetist who regularly worked at the hospital in question and another public hospital nearby [12]. In the ensuing weeks, the active search for other potentially infected patients led to the detection of a large outbreak possibly involving hundreds of patients, all of them related to the activity of this medical professional. Almost all the patients had been previously treated in the two hospitals where the anesthetist practiced regularly. The epidemiological evidence gathered during the first 3 months of investigation confirmed the initial suspicions of the existence of an outbreak linked to the professional activity of this particular anesthetist. The epidemiologists examined the association of over 66,000 people who had undergone surgery in the 2 hospitals with the usual risk factors for infection in surgical procedures: surgeon, surgery room, type of surgery, anesthesiologist, type of anesthesia, and so on. The only significant factor (adjusted OR 28.5, 95% CI 9.83 to 82.59) was this anesthetist (H. Vanaclocha, DGSP-Conselleria de Sanidad, Generalitat Valenciana, and F. Martinez, Centro Nacional Epidemiología, SPAIN). Furthermore, of the initial 197 cases considered to be included in the outbreak, 184 had been anesthetized by him. No additional links were found among these patients, nor the ones who were included later on. Other hypotheses for the possible sources of infection were discarded on the basis of the epidemiological evidence.

Public health authorities and the judge in charge of the corresponding epidemiological and judicial investigations requested our expertise in evolutionary biology in order to (i) check whether the suspected source was actually responsible for the outbreak, (ii) ascertain which patients had been infected from a common source and could be considered as included in the outbreak and who had been infected from alternative sources, (iii) discard alternative sources or the existence of different but simultaneous outbreaks, (iv) determine the duration of the outbreak, (v) date the time of infection for each patient involved in the outbreak, and (vi) determine the date of infection of the source.

Here, we present a molecular and evolutionary epidemiological analysis of this outbreak based on HCV sequences obtained from the presumed source and the patients. The analysis was used to discriminate who out of the potential victims had actually been infected by the common source, to provide an individual assessment of the likelihood of this assignment, and to obtain an estimate of the date of infection for each patient. These results helped the court to convict the anesthetist of professional malpractice leading to the infection of 275 of his patients.

## Results

We received serum samples of 322 HCV-1a and 290 HCV-1b positive patients who had been in contact with the presumed source (PS) or had been attended at any of the hospitals where he worked regularly. Once we determined that the PS carried only HCV-1a (see below), HCV-1b samples were not processed further. We also determined the sequences in 44 samples from persons infected with HCV-1a in the city of Valencia who were not related to the outbreak, based on the epidemiological evidence, and whose sera had been stored at −80°C in local hospitals. These samples were used as local controls. The nucleotide sequence of two HCV genome regions was determined after reverse transcription (RT) of viral RNA into DNA followed by hemi-nested polymerase chain reaction (PCR). A 229-nucleotide (nt) fragment of the *NS5B* gene, which encodes the RNA-dependent RNA polymerase of the virus, was analyzed by direct Sanger sequencing of PCR products. This region corresponds to a relatively conserved portion of the viral genome [13] and the method used provides only a consensus sequence of the whole spectrum of genetic variability present in each sample. The second region we analyzed was a 406-nt fragment encompassing the C-terminal end of the envelope-1 and the N-terminal of the envelope-2 glycoprotein (E1-E2 region). This much more variable region of the HCV genome was analyzed by sequencing at least ten viral inserts cloned in recombinant plasmids derived from RT-PCR products per patient.

**Figure 1:**
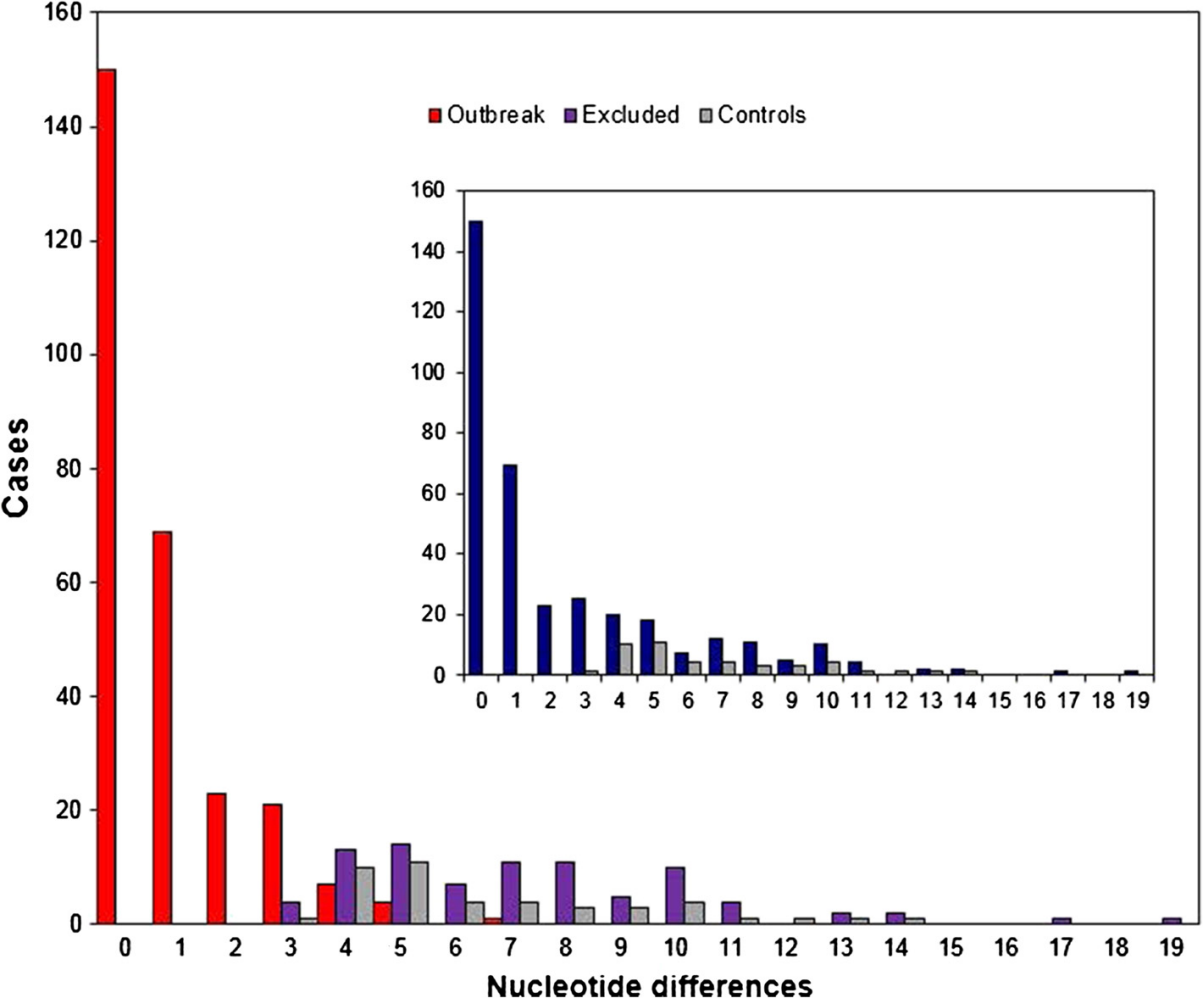
**Distribution of differences in the NS5B region.** Sequences were compared with a 229-nucleotide (nt) fragment of the NS5B gene derived from the presumed source (PS). The graph shows the distribution of nucleotide differences (Hamming’s distance) for sequences derived from patients included in the outbreak (red bars), from patients excluded (dark purple) from the outbreak, and from local controls (grey). The inset shows the same distribution for putative outbreak samples (dark blue) and local controls (gray) before the former were divided into included and excluded from the outbreak.

We obtained 320 sequences of the NS5B region from HCV-1a samples and 44 from local population controls. The nucleotide sequence of each sample was compared to that of the PS and the distribution of differences (Hamming distance) was determined (Figure 1). The *NS5B* sequence obtained from the PS sample corresponded to HCV subtype 1a. To further check that the HCV present in the PS was only of subtype 1a, PCR products of the NS5B region were cloned and subsequently sequenced individually. All the clones analyzed (n = 25) corresponded to subtype 1a, which led us to discard the possibility of a dual infection (HCV 1a/1b) in the PS. As a consequence, no further analyses were performed on HCV-1b sequences and the corresponding patients were excluded from the outbreak investigation.

Sequences derived from samples putatively included in the outbreak presented from 0 to 19 differences from the PS sequence in this 229-nt region (mean = 2.37, SD = 3.31), whereas the number of differences between the controls and the PS sequence ranged from 3 to 14 (mean = 6.61, SD = 2.75). We observed a large number of cases (n = 150) of perfect identity to the PS sequence in the analyzed fragment and 69 and 23 sequences with only 1 and 2 differences, respectively, leading to a highly asymmetric distribution of differences from the PS in the outbreak sequences. This was in sharp contrast with the distribution obtained for the control samples (Figure 1). Hence, this initial analysis already identified the existence of a large number of samples harboring HCV of subtype 1a more closely related to the PS than to the local controls, although it did not provide enough resolution to clearly separate these 2 populations: 118 samples presumably from the outbreak presented a number of differences that overlapped the range spanned by the control samples.

**Figure 2:**
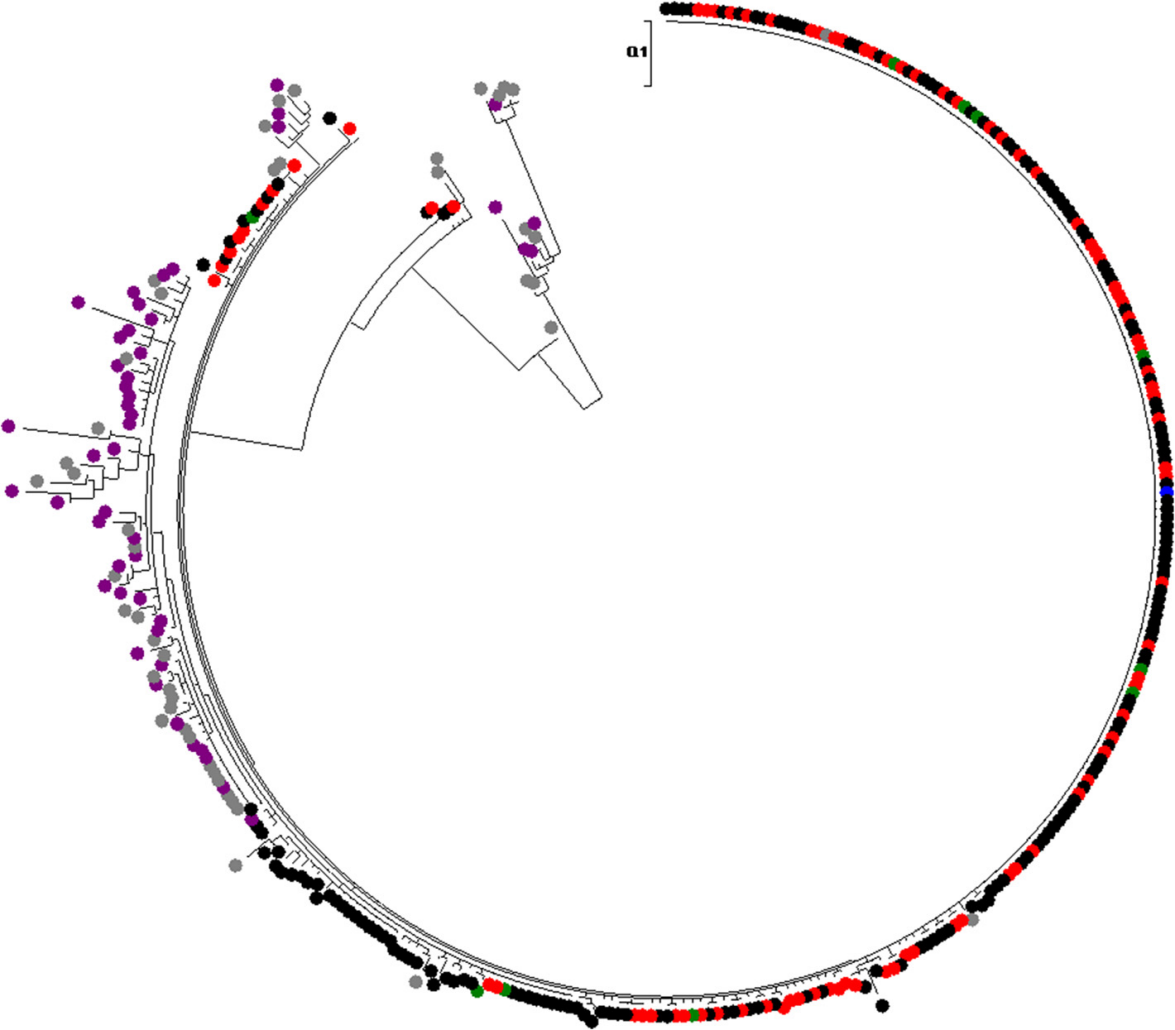
**Neighbor-joining tree obtained with the NS5B-region sequences of hepatitis C virus (HCV)-1a samples analyzed in this study.** Color codes: outbreak sequences are in black, red, and green (see legend to Figure 3), excluded from the outbreak are in dark purple, and local unrelated controls are in gray. The presumed source (PS) sequence is shown in blue. No clade was found with bootstrap support higher than 70%.

Neighbor-joining and maximum likelihood phylogenetic trees obtained from the NS5B sequences failed to group all the control samples in a monophyletic group (Figure 2). Furthermore, none of the nodes in the phylogenetic tree received a bootstrap support higher than 70% by either phylogenetic reconstruction method. This is not surprising considering that NS5B evolves much more slowly than other regions in the HCV genome (especially the E1-E2 region) and the relatively short sequence length analyzed. As a consequence, the phylogenetic signal in this region was too low to reliably separate the local controls, the patients infected from a common source and the patients infected from alternative sources.

We obtained 4,184 cloned sequences from the E1-E2 region of the HCV genome. For the PS, we obtained 134 cloned sequences from the only sample available (taken on 12 February 1998). Under the Spanish legal system, it was not possible to obtain another sample from the suspected source of the outbreak and we intended to estimate as accurately as possible the genetic variability of the virus in the PS of the outbreak. Of the remaining clones, 3,597 sequences corresponded to 321 samples initially considered to be in the outbreak, and 453 sequences were from 42 local controls. It was not possible to obtain E1-E2 cloned sequences from two local controls and one putative outbreak patient. Excluding the PS, we set a goal of ten sequences per sample. The average number of cloned sequences per patient actually obtained excluding the PS was 10.77, ranging from 6 to 20 for the outbreak samples and from 10 to 30 for the controls. Deviations from the initial goal were due to the occasional independent referral of several samples from the same patient. Once their coincident origin was verified, they were considered as one single sample in the ensuing analyses.

The 134 sequences derived from the PS were not identical to each other, presenting 28 different haplotypes and an average of 2.07 differences in pairwise comparisons. These sequences clustered in 2 groups, with 127 and 7 sequences, respectively, which differed in their genetic variability. The large group included 22 different haplotypes and was less variable (haplotype diversity, H = 0.474; nucleotide diversity, π = 0.0019) than the smaller group (6 haplotypes, H = 0.952, π = 0.0075). The average nucleotide divergence between the two groups was 0.0301.

**Figure 3:**
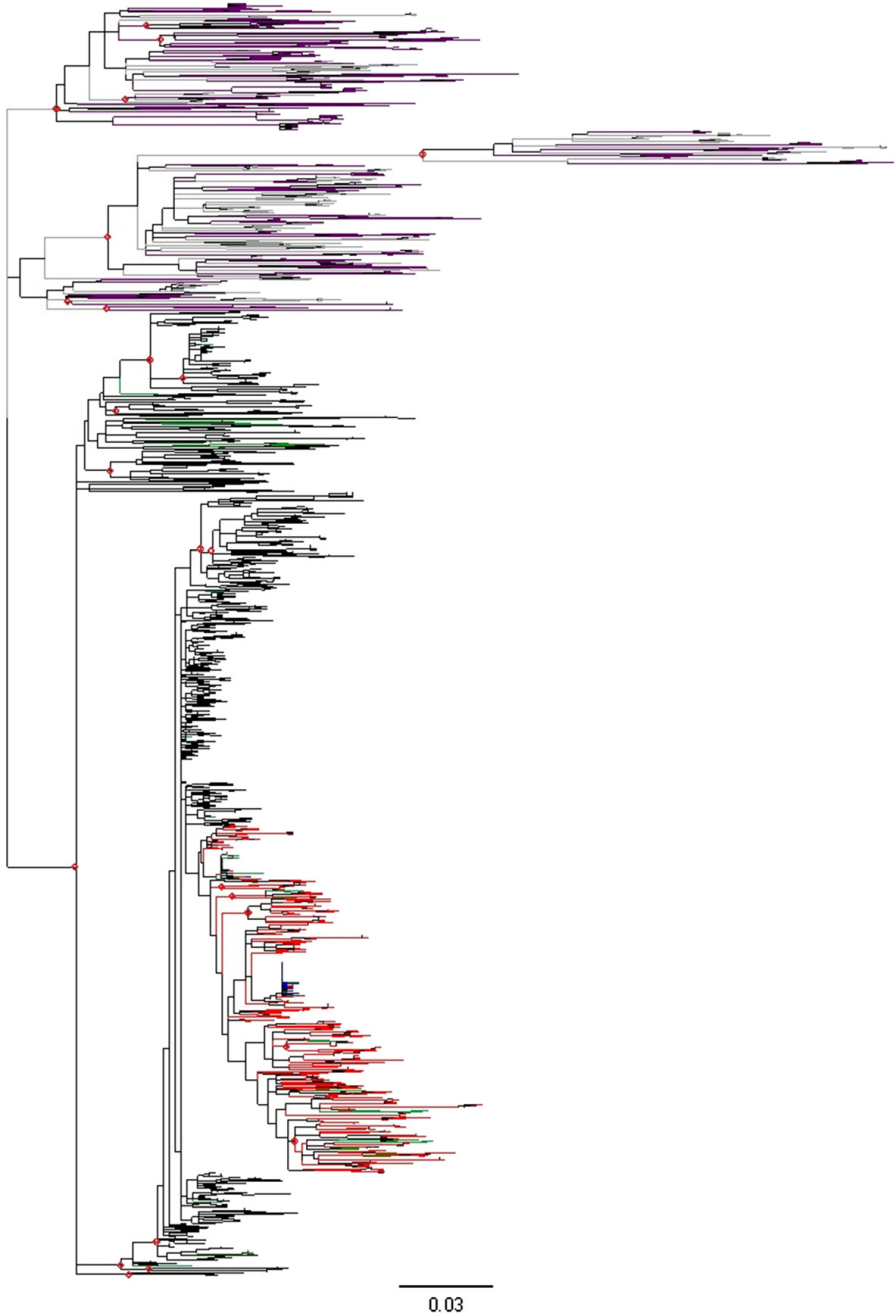
**Maximum likelihood tree for cloned sequences in the E1-E2 region.** The tree includes 4,184 sequences from a 406-nucleotide (nt) fragment of the E1-E2 region including hypervariable region (HVR)1 and HVR2. Sequences were obtained from patients included in the outbreak (274 patients, 3,038 sequences), patients excluded from the outbreak (47 and 559, dark purple), local controls (42 and 453, gray), and the presumed source (PS; 134 sequences, dark blue). Sequences and branches in the monophyletic clade defined by all the cloned sequences from the PS are labeled in red. Sequences from polyphyletic samples with some representatives in the clade delimited by the PS sequences and others outside it are labeled in green. Relevant nodes with bootstrap support larger than 90% are indicated by red dots. Further details can be obtained in the annotated treefile provided as Additional file 2.

After the multiple alignment was obtained (Additional file 1), we derived neighbor-joining and maximum-likelihood phylogenetic trees for the 4,184 cloned sequences. Both trees shared a highly supported internal branch (bootstrap support = 100% and 96% after 1,000 replicates in the NJ and ML reconstructions, respectively), which was used to define the patients included in the outbreak: sequences from 274 patients were grouped with the sequences from the PS, while the second group included all the sequences derived from the local controls and sequences from 47 patients initially considered to belong to the outbreak (Figure 3 and Additional file 2).

Contrary to other cases of molecular epidemiological analyses of outbreaks produced from one single individual infected with an RNA virus (for example, [7]), paraphyly of the source sequences was not the hallmark of the outbreak and could not be used to define the extent of the outbreak or which patients had actually been infected by the PS. The minimum clade encompassing all the cloned sequences derived from the PS (blue branches in Figure 3 and Additional file 2) included 1,011 sequences from 97 different patients (red branches in Figure 3 and Additional file 2). Under the assumption of paraphyly, this would be the group of outbreak patients. However, not all the cloned sequences of ten of these patients were included in this clade. A total of 52 sequences obtained from these 10 patients were included in other groups, external to the clade defined by the cloned sequences from the PS (green branches in Figure 3 and Additional file 2). We considered that these sequences were also derived from the same initial population although no representative of these variants had been found in the sample analyzed from the PS (further discussed below). As a consequence, we continued evaluating the minimum clade that included all the cloned sequences derived from to the PS and all the patients for which at least one clone was related to those from the PS or from a patient related to the PS, as described above.

This procedure led to a larger monophyletic cluster that included 3,038 sequences from 274 patients and 134 sequences from the PS (black, red, green and blue branches in Figure 3 and Additional file 2). This cluster was highly supported in the neighbor-joining and maximum-likelihood phylogenetic reconstructions (Figure 3 and Additional file 2) and it included no sequences from the local controls. Sequences from the 42 local controls (gray branches in Figure 3 and Additional file 2) conformed a separate cluster, also with high bootstrap support, that also included 640 sequences from 47 patients initially suspected to belong into the outbreak and who were correspondingly removed from this category (purple branches in Figure 3 and Additional file 2) and considered to have been infected elsewhere despite their epidemiological association to the PS. The assignment of each individual presumably related to the outbreak to one category or the other was further tested under a more rigorous statistical framework.

For each individual we considered two alternative hypotheses: either the patient had been infected by the PS and should be included in the outbreak, or had been infected from a different source and should be excluded from the outbreak. In the former case, sequences derived from the case should group with those in the outbreak whereas in the latter they should group with the controls and the excluded patients. For each alternative, we obtained the likelihood of the corresponding phylogenetic tree [14]. For sequences in the outbreak group, as determined by the strongly supported interior branch described above, the alternative hypothesis was evaluated by computing the likelihood of the phylogenetic tree obtained by moving all the sequences obtained from the corresponding sample to the base of the clade defined by control and non-outbreak samples (Additional file 3). Similarly, the alternative phylogenetic tree for the excluded samples, which corresponded to their assignment to the outbreak group, was obtained after shifting the corresponding sequences to the base of the outbreak-defining clade. The ratio between the two likelihoods is a measure of the relative support provided by these data to each hypothesis and, therefore, can be easily translated into an expert forensic evaluation [15].

For the cases assigned to the outbreak according to the phylogenetic reconstruction described above we obtained likelihood ratios (LRs) in the range 1.051 to 6.622 × 10^95^. The lowest values corresponded to the patients whose sequences, although included in the outbreak, were the closest to the control and non-outbreak group. For these patients, the change in likelihood was minimal, since the topologies used in the test were devised as the most favorable for the accused (the PS), thus minimizing the probability of incorrect assignment of patients to the outbreak. For most patients (n = 240), the LR value was higher than 10^5^, which represents a very strong support for their individual assignment to the outbreak group. Similarly, support values for those patients who were finally excluded from the outbreak group, given by the LR between this hypothesis and the alternative of their inclusion in the outbreak, ranged between 1.330 and 4.408 × 10^84^, also providing very high support for their lack of association to the outbreak. Based on these results, 47 patients who were initially considered to be part of it because of their epidemiological links to the PS were excluded from the outbreak. The court accepted this argument and removed these patients from the court process.

The phylogenetic analysis was consistent with the epidemiological evidence in identifying the PS as the source of the outbreak. In the next step of our analysis we assumed that the PS was indeed the source and used the Bayesian method with constant population size and relaxed molecular clock implemented in BEAST (‘Bayesian Evolutionary Analysis by Sampling Trees’) [16] to estimate the infection dates of the 274 patients included in the outbreak. For each patient, E1-E2 cloned sequences were used to establish the time to the most recent common ancestor (MRCA) to the closest group of E1-E2 cloned sequences derived from the PS. Apart from the information on the dates when samples had been obtained, we used the known infection dates of 24 patients in the outbreak [17]. These patients were chosen because they had had contact with the PS only once, at a known date, and had tested negative for HCV before that date and positive afterwards. Consequently, their sequences were used to calibrate the molecular clock estimates for the MRCA of each outbreak patient and the PS (Additional file 4).

Estimates of divergence for each outbreak patient from the PS ranged between January 1987 and April 1998. These values correspond to the medians of the 95% Bayesian highest posterior density (HPD) for each estimated date to the MRCA with the PS. When the upper and lower ends of each interval were considered, the latter date is not contradictory with the detection of the outbreak in February 1998. The estimated time of infection for the PS was June 1988, with 95% HPD intervals ranging from August 1984 to October 1991, and it was thus compatible with the earliest estimate for the date of infection of an outbreak patient. The estimated time of infection for each patient was compared to independently derived estimates by the prosecution during the trial. These were based on hospital records and other documents, and did not consider any sequence-based information.

**Figure 4:**
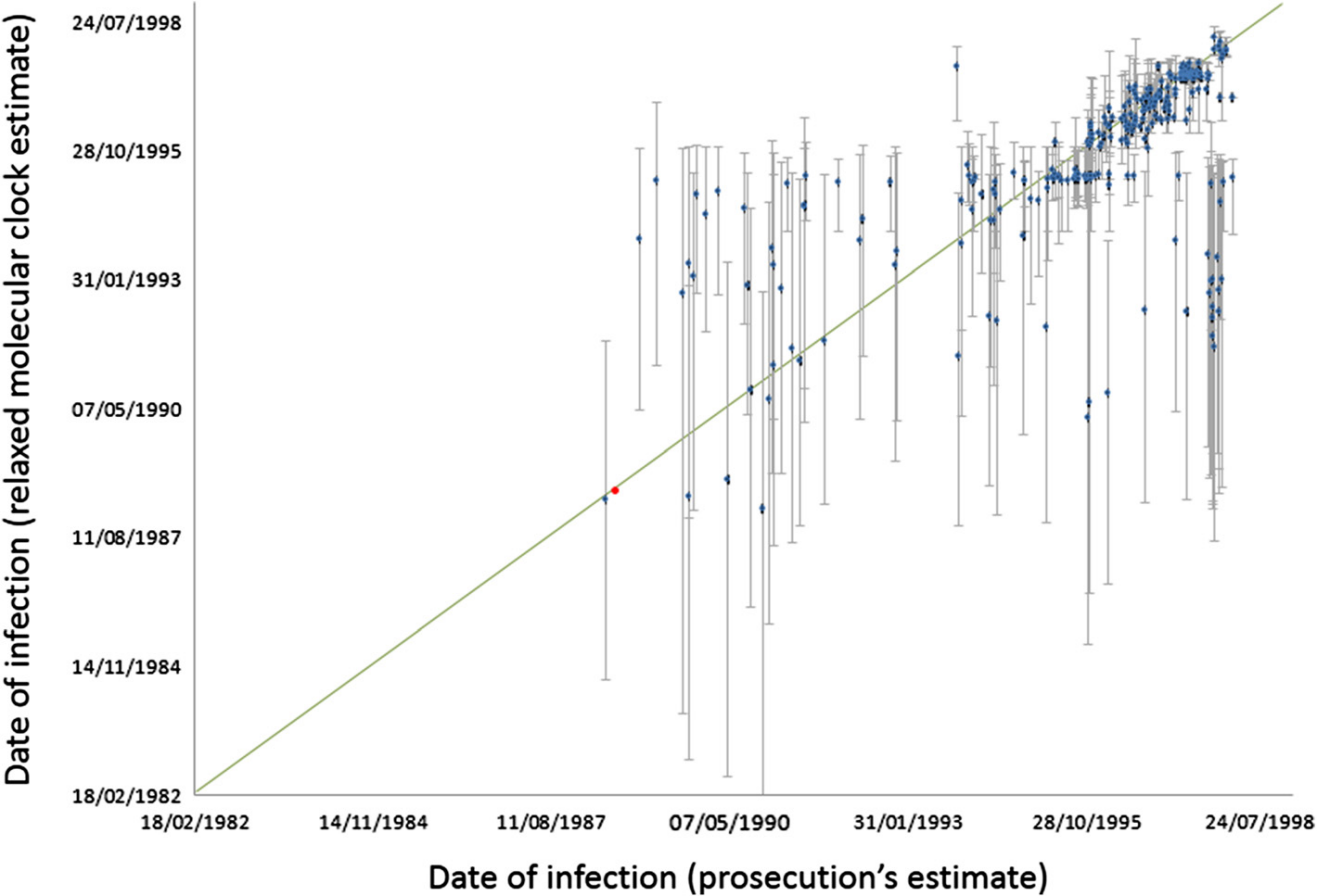
**Inferred dates of infection.** Each point shows the median estimate and the 95% highest posterior density (HPD) region estimated using a relaxed molecular clock model, as detailed in the text. Prosecution estimates were taken as the most likely date according to the prosecutor’s final report. The red dot represents the inferred date of divergence of the sequences derived from the presumed source from the common ancestor of control sequences. No estimate of the infection date for the presumed source (PS) was given by the prosecution.

A comparison between these 2 estimates for each patient is shown in Figure 4, where it can be observed that in 176 cases (65%) the HPD interval for the Bayesian estimate of infection included the most likely estimated date given by the prosecution to the court, based on documents and testimonies from several sources, but in some cases alternative dates were possible (Additional file 5: Figure S1). Most differences between the two estimates corresponded either to the oldest infections or to the most recent ones. The former can be explained by lack of appropriate calibration samples, since the earliest date in this group corresponded to a patient infected in March 1995. It should be noted that estimates correspond to divergence from the last common ancestor and these should precede the actual date of transmission from the source to the recipient. The underestimation of the date of some recent infections may stem from the still insufficient sampling of the PS viral population. These patients were likely infected by a subgroup of PS variants that were not represented in the PS sample used in our analyses. If so, the estimates would correspond to the time of divergence to the PS variants included in our sampling, not actual infection dates, and would predate them.

## Discussion

The rapid rate of evolution of pathogenic RNA viruses represents an important problem for the design and application of efficient therapeutic and vaccination strategies. However, it also represents an extraordinary opportunity to observe evolution in real time [18]. In the case described here, we used the fast evolutionary rate of HCV to disentangle a large and complex transmission process from a single source to almost 300 recipients spanning over a decade, a period during which the infecting viral population underwent evolutionary changes itself. The process was further complicated by two additional issues: a large number of potentially affected patients, several of which might have been infected by HCV from alternative, unidentified sources, and the need to provide individual rather than population-based statements about the likelihood of having been infected or not from the presumed source in a court setting.

The main difficulty encountered in the interpretation of the expert testimony by the court (judges, prosecutors, defense and accusation attorneys, and so on) was their lack of familiarity with evolutionary theory and processes, especially when these occur in such short timespans as those involved in this case. The commonly held notion is that evolution is a process that occurs over long periods of time and that it can only be observed in scales of thousands or millions of years, but not in months. The need for variation in order to apply molecular evolution methods is at odds with the search for identity between the genetic markers recovered in a crime scene and those of the potential culprits, or between the offspring and the alleged father once maternal markers have been considered. These are the most common type of data and situations in which DNA profiling is brought to courts and, as a consequence, what most people not familiar with evolutionary theory expect to find in this type of expert testimony is a perfect match between the parental and the offspring viruses indicating a direct relationship between the source and the recipient. Molecular epidemiology analyses of rapidly evolving microorganisms have to be framed within evolutionary theory since only this provides the necessary concepts to ascertain proximal and distal relatedness from the observed genetic variation [18,19]. These principles have been successfully applied in previous cases of HIV transmission brought to courts [4,7,20,21] and to many other cases of HIV and HCV transmissions that did not lead to legal investigations [2,5,22-24]. However, none of these involved the investigation and analysis of a large number of potential recipients of the virus from the same source, which continued evolving during the long period in which infections occurred in the case considered here.

Previous cases of large HCV outbreaks affecting hundreds of persons [25,26] were caused by contamination of blood derivatives by a single donor. As a consequence, all the infected patients received a very similar sample of the virus population present in the corresponding sources at the moment of blood donation. In these cases the common ancestry could be traced to a relatively homogeneous initial sample, which does not evolve until transferred to a new host that, with no doubt, facilitates the identification of a common origin of the outbreaks. In the case reported here, the sequences recovered from the outbreak patients correspond to different inoculums from a viral population that had been evolving continuously under the pressure of the immune system of the source for about 10 years, the time since the infection of the anesthetist from an unknown source until the detection of the outbreak and the cessation of his professional activity. Evolution in the source during the long period along which transmissions occurred further combined with evolutionary changes in each infected patient have produced a wide array of viral sequences whose common ancestry could only be inferred after taking into account the whole spectrum of variants generated during the process.

In addition, there is mounting evidence that compartmentalization occurs in individuals infected with HCV [27-33] and the analysis of E1-E2 cloned sequences in patients related to this outbreak further supports this possibility. Compartmentalization refers to the microevolutionary processes of viral populations occurring in separate tissues and organs of an infected individual that might lead to significant differences among subpopulations within that individual. HCV is transmitted through blood, but the blood is not the primary reservoir for the virus in the infected body. In fact, although the liver is the main organ infected by HCV, this virus has been shown to infect and replicate in other tissues that will eventually contribute to the HCV population circulating in the bloodstream.

In addition to compartmentalization, an additional process has likely contributed to generate the complex pattern of variation in the viral populations obtained from the PS and the infected patients. Several features of HCV populations such as re-emergence of variants after treatment or lack of association between viral features and response to treatment or disease progression have been recently interpreted in light of within-patient dynamics of the virus [34-36]. These analyses have revealed the coexistence of relatively divergent lineages within chronically, but also acutely, infected patients that are not necessarily present simultaneously in plasma. Given that HCV is mainly transmitted through blood, which actual variants are transmitted from the same source to different recipients can vary depending on the viral population circulating at the moment of infection. Although these populations have been characterized in serial samples from the same patients usually a few weeks or months apart, it is evident that this same process may explain differences observed on larger timescales. For instance, patients receiving HCV-infected sera from a common blood donor were shown to harbor different viral subpopulations that were still present a few months/years after infection and that evolved into well differentiated clades a few years later [37]. Hence, it is possible that compartmentalization and intra-patient fluctuations of genetic variants caused a departure from the paraphyly model postulated to characterize the populations of donor and recipients in viral transmission cases [7] in the case of the E1-E2 cloned sequences analyzed here, if different patients received different viral inoculums depending on the actual populations circulating in the blood of the donor at the time of infection. Naturally, further independent evolution within each new host would enhance any differences at the time of infection. In this case, it is also necessary to consider the long period of infection of the PS, which further facilitates differentiation of viral subpopulations within and among compartments.

Paraphyly of source sequences is usually invoked to determine the direction of transmission [7]. As discussed previously, not all the sequences derived from patients considered to be in the outbreak group were included in the monophyletic group defined by the sequences derived from the PS. Our preferred explanation for this observation has been discussed in the previous paragraphs, but it could be hypothesized that the PS had been infected by some of his patients and that he had subsequently infected others. In this case, the PS would be an intermediate link in a transmission chain and not the central hub in a large series of transmission pairs. The reasons for discarding this alternative possibility were as follows. There are two ways in which some patients have non-monophyletic sequences, with one group of them included in the monophyletic group defined by the common ancestor to the sequences of the PS and the other in separate, but nevertheless related, groups. One is that each of these patients had been coinfected by the PS and by an alternative source. The other is that there had been only one infection from the PS but the infecting viruses were already heterogeneous and relatively divergent in the source so that differences between the two groups within these patients would lead to the observed pattern. Our main argument against the first possibility is that a secondary common source (in fact several sources) would have to exist that might explain their grouping with other sequences from patients whose only known (and common) risk for HCV infection was determined in the epidemiological investigation to be the physician. How can we explain, and prefer, the second possibility? Firstly, the already mentioned epidemiological linkage was very strong and it led us to prefer any possibility with one single infection rather than alternatives with two or more infections for which no evidence was ever found. Secondly, we have already discussed how compartmentalization within the PS and viral evolution within him over a 10-year period can explain the observed pattern without any need for unsupported claims of other processes.

We must emphasize that the methodology used in this work is appropriate for testing hypotheses derived from previous, independent investigations. In this case, the epidemiological enquiry revealed a highly likely source for the outbreak and our goal was to test this hypothesis as rigorously as possible. Given the size of the outbreak and the prevalence of HCV infection in our country, it was a likely possibility that not all the outbreak-related cases had been infected by the same source, and this was actually proven for 47 infections. Similarly, the direction of the infection, although strongly suggested from the global tree from the E1-E2 region shown in Figure 2 to be from the PS to the patients and especially for the smaller clade encompassing all the sequences derived from the PS, was also grounded in our previous knowledge of epidemiological investigation, with only 1 common link for the 275 patients included in the outbreak (namely, their anesthetist). This implied a particular direction of the infections that is compatible with the non-molecular evidence and also, as detailed in the preceding paragraphs, with the sequence data obtained once knowledge about the intrapatient dynamics of chronically infecting virus such as HCV is taken into account. As established by Evett and Weir [15], and further discussed below, the scientific expert must give a quantitative estimate of the relative support that the data in his/her domain provide to each hypotheses (innocence or culpability), which should be considered along with additional pieces of evidence gathered from other sources of investigation.

We agree that this is an unusual and also unexpected pattern for a single viral outbreak, but this is so not only at the molecular level. As commented previously, this was an unprecedented outbreak for an RNA virus capable of establishing a chronic, asymptomatic infection, which certainly contributed to its long duration and large number of infected patients. A similar case, also involving a medical professional spanning several years and in different geographical locations with possibly dozens of infected individuals from the same source, has been recently reported in the USA [38]. If similar circumstances to those exposed in the case described here concur in this new case, we anticipate that similar patterns at the molecular epidemiological level will be observed. The actual procedure on how the anesthetist infected so many of his patients and the reasons for doing so are naturally out of the scope of this report. Nevertheless, the court sentence established that the anesthetist had used for himself the same materials and drugs employed with his patients, and that these uses were previous to the corresponding medical acts (anesthesia, painkilling, and so on). No evidence was established in the trial about him knowingly infecting the patients or having information about his own HCV-positive status.

Molecular phylogenetic reconstructions have become increasingly popular over the last few decades mainly as a result of easy and cheap access to gene, genome and other large-scale sequencing methods and to the development of user-friendly platforms for the analysis of sequence data. However, the direct application of these methods in forensic analysis has to be made even more cautiously than for general scientific enquiries given the potentially serious consequences of a wrong inference or conclusion in a criminal setting. Some of the problems arising in the inference of transmission chains or outbreak sources on the basis of molecular phylogenetic analyses have been commented on elsewhere [39,40]. For instance, the use of an inappropriate genome region can lead to erroneous inferences, as we observed in the analysis of the NS5B region in this outbreak.

The development of next-generation sequencing methodologies for fast and accurate analysis of viral populations [41-43] has already led to its application in a case of HCV transmission [44] and it might become eventually a routine technique in this setting [45], thus overcoming some of the limitations derived from the strategy of cloning and sequencing PCR products that we had to use in this work. Similarly, recent developments in algorithms and computer speed and capabilities [46,47] may also allow the application of more rigorous and encompassing phylogenetic analyses than those we were able to apply to these data, such as obtaining global estimates for the dates of infection for all the patients in the outbreak or using all the sequences available from each patient to obtain those estimates.

Beyond methodological issues on the use of one or other marker for molecular epidemiology or the most appropriate model for phylogenetic inference, the application of Evett and Weir’s [15] procedure and the extension that we propose in this work are highly recommendable. According to Evett and Weir, scientific experts must inform on the likelihood of the observed data under the different hypotheses and in light of other evidences available. This is especially relevant in the determination of outbreak sources because other alternative routes of infection, such as secondary infection from a primarily infected patient or infection from a third unidentified source, have to be ruled out. In the case described here, these possibilities were discarded in the course of an extensive epidemiological investigation and in our testimony in court we simply provided an evaluation on the likelihood of the sequences derived from each patient suspected to have been infected from the presumed source. Similarly, and in this situation this was notably important in terms of preserving the assumption of innocence unless otherwise proven, we were able to discard from the outbreak a group of 47 patients who complied with all the epidemiological criteria for inclusion in the outbreak but whose viruses had a higher likelihood of having a different origin than those from the outbreak.

## Conclusions

The combination of recent developments in molecular evolutionary analysis and the statistical framework developed for the forensic study of nucleic acid samples has allowed us to incorporate molecular epidemiology, with its natural components of molecular phylogenetics and population genetics, into the realm of forensic analysis. Despite tremendous progress in the prevention of infections by HCV and other blood-borne viruses in the last decades, nosocomial and other transmissions related to medical procedures still occur [48,49]. Here, we have provided an adequate methodology for the rigorous testing of alternative hypotheses used in the epidemiological and forensic analysis of these infections in a real, complex and massive case.

## Methods

### Source of INNO-LiPA, HCV II, Innogenetics, Ghent, Belgium-positive samples

Persons suspected to have been exposed to HCV infection by the PS were actively searched for by the regional Public Health Services. Serum samples from these patients and unrelated local controls were tested for HCV infection by enzyme-linked immunosorbent assays (ELISA) and further confirmed by HCV-specific RNA amplification. Determination of HCV genotype was performed by reverse hybridization of PCR amplicons of the 5′ non-coding region (INNO-LiPA HCV II, Innogenetics, Ghent, Belgium).

### Samples and patients

Samples were collected as contemporarily as possible. To avoid cross-contamination, each sample was physically isolated during RNA purification and gloves were changed frequently during all procedures. Different laboratories and devices were used for RNA isolation, DNA amplification, cloning and sequencing, and negative controls were included at all steps.

### RNA extraction and reverse transcription

Viral RNA was purified from 200 μl of serum using the High Pure Viral RNA Kit (Roche Diagnostics GmbH, Mannheim, Germany). RT was carried out in a 20-μl volume containing 5 μl of eluted RNA, 5 × RT buffer, 500 μM of each dNTP, 1 μM of hexamers, 100 U of M-MLV Reverse Transcriptase (Promega Corp., Madison, WI, USA), and 20 U of Recombinant RNasin® Ribonuclease Inhibitor (Promega). Reaction mixtures were incubated at 37°C for 60 minutes, followed by 2 minutes at 95°C.

### Amplification and direct sequencing of the NS5B region

Direct sequences of PCR products were obtained for a 337-nt fragment of the NS5B gene, although we used only a 229-nt subfragment corresponding to the sequence of the PS initially determined by another laboratory. PCRs were performed in a 50-μl volume containing 5 μl of RT product, 100 μM of each dNTP, 200 nM of each primer, and 2.5 U of *Taq* polymerase (Amersham Biosciences, Piscataway, NJ, USA). Oligonucleotides used for amplification and direct sequencing of NS5B region were: 5′-TATGATACYCGCTGYTTYGACTC-3′ (sense) and 5′-GTACCTRGTCATAGCCTCCGTGAA-3′ (antisense). Direct sequencing of purified PCR products was performed on an 8-μl volume, including 1.0 μl of DNA, with the ABI PRISM BigDye Terminator Cycle Sequencing Ready Reaction kit in ABI 377 or ABI 310 automated sequencers (Applied Biosystems, Foster City, CA, USA). Both strands were assembled using the Staden Package. Sequences were verified and deposited in GenBank with accession numbers FR670793-FR671156.

### Amplification, cloning and sequencing of the E1-E2 region

A 406-nt fragment of the E1-E2 region was amplified by nested PCR. The first amplification was performed in a 100-μl volume containing 10 μl of cDNA, 10 × PCR buffer, 100 μM (each) dNTP, 400 nM (each) oligonucleotide and 2.5 U of *Pfu* DNA polymerase (Stratagene, La Jolla, CA, USA). Oligonucleotides used for amplification of E1-E2 region were: 5′-CGCATGGCATGGRATATGAT-3′ (sense), 5′-GGAGTGAAGCARTAYACYGG-3′ (antisense), 5′-GGRATATGATGATGAACTGGTC-3′ (nested sense). Both primary and nested PCR were performed with the following thermal profile: 94°C for 3 minutes; 5 cycles at 94°C for 30 s, 55°C for 30 s, and 72°C for 3 minutes; 35 cycles at 94°C for 30 s, 52°C for 30 s, and 72°C for 3 minutes; and a final extension at 72°C for 10 minutes. Amplification products were purified and directly cloned in *Eco*RV-digested pBluescript II SK(+) phagemid (Stratagene). Plasmid DNA was purified with the High Pure Plasmid Isolation Kit (Roche). Recombinant clones were sequenced by the use of KS and SK oligonucleotides (Stratagene) and the same procedure described above for the NS5B region but using 5 μl of purified cloned DNA. The obtained sequences have been deposited in GenBank with accession numbers FR671450-FR675633.

### Molecular phylogenetics and evolutionary analysis

Multiple alignments were obtained with ClustalW 1.82 [50]. Estimates of Hamming’s distance between each NS5B sequence and that obtained from the PS were obtained with MEGA 2.0 [51]. Due to computational limitations at the time of the initial analysis (1999 to 2001), an initial phylogenetic tree was obtained with the neighbor-joining algorithm [52] using Kimura-2P distance [53] for pairs of sequences with program ClustalW. Subsequently, the same sequences were used with program PHYML [54] to obtain a maximum likelihood tree using the GTR + I + G model determined by Modeltest [55] using AIC [56]. Support for internal nodes was evaluated with 1,000 bootstrap replicates [57].

### Statistical analysis of competing hypothesis

We adopted Evett and Weir’s [15] method for the use of genetic analyses in forensic settings using a Bayesian framework. This method is consistent with the judicial procedures in Spanish courts. Briefly, these authors indicate that scientific experts’ contribution to forensic analyses of genetic information should restrict to the evaluation of the available genetic data in light of the two competing hypotheses, those of the prosecution and the defense. The jury or judges must integrate that information, ideally in a numerical way, with the information provided by other types of evidence. Formally, this is accomplished by computing the ratio of two posterior probabilities, those of the prosecution and the defense proposals conditioned on all types of evidence for whose computation it is necessary to use the corresponding priors, here defined as the probability of each hypothesis in light of the non-genetic evidence (*I*), and the likelihood of the genetic data (*G*) conditioned on the non-genetic evidence and each hypothesis (*H*_*p*_ and *H*_*d*_):

$$\frac{\mathrm{Pr}\left(H_{p}\left|G,I\right)\right)}{\mathrm{Pr}\left(H_{d}\left|G,I\right)\right)}=\frac{\mathrm{Pr}\left(G\left|H_{p},I\right)\right)}{\mathrm{Pr}\left(G\left|H_{d},I\right)\right)}\times \frac{\mathrm{Pr}\left(H_{p}\left|I\right)\right)}{\mathrm{Pr}\left(H_{d}\left|I\right)\right)}$$

In the context of molecular phylogenetic analysis applied to forensic epidemiology, the two competing hypotheses correspond to the assignment of a given patient to the group of patients infected from a common source or to that of control sequences in which there is no common source of infection. The evaluation of competing phylogenetic hypotheses under a maximum likelihood framework directly provides their likelihood ratio (LR), thus allowing us to derive individual estimates of the support from the sequence data to the two relevant alternative proposals: (i) a given patient was infected with HCV in the outbreak, and as a consequence viral sequences obtained from him that share a closer common ancestor with those obtained from the source and other patients in the outbreak, or (ii) the patient was infected from other source not linked to the outbreak, so that his or her viral sequences are evolutionarily closer to those from controls and patients excluded from the outbreak than to sequences from outbreak patients. Hence, it is possible to provide quantitative criteria for deciding between alternative possibilities to the jury or those in charge of taking a judicial decision.

Patients were included or excluded in the outbreak depending on their grouping in the phylogenetic tree of the E1-E2 region sequences. The likelihood of each alternative topology was obtained with program fastDNAml [58], allowing it to optimize branch lengths for each case. The LR for each patient was computed considering the whole data set: the PS, the controls, the excluded patients, the remaining patients in the outbreak and the target patient. Hence, values are referred to the change in likelihood of the E1-E2 cloned sequences tree when the sequences of each patient were placed in the node delimiting the controls and non-outbreak clade (for outbreak patients) or the outbreak clade (or non-outbreak patients).

### Estimation of infection dates in the Bayesian Markov chain Monte Carlo (MCMC) framework

The relaxed molecular clock model [59] implemented in BEAST [16], under constant population size (more complex models did not increase the accuracy of the estimation; [17]) and using the GTR + I + G model, was used to estimate the infection dates of the 274 patients included in the outbreak and for whom E1-E2-region cloned sequences were available. Sequences from 24 patients for which the infection dates were known with certainty were employed to calibrate the clock [17]. The method used here for the estimation of infection dates is an extension of the approach we used previously in a validation study that tested the accuracy of estimation of infection dates in this outbreak using the Bayesian approach [17] for the 24 patients whose infection dates were known. The calibration assumed that the infection dates for these 24 patients corresponded to the time of the most recent common ancestor between the sequences of a given patient and the appropriate PS population (see above). In all the analyses, the tips were dated with the corresponding sampling times for all the patients, controls and the PS. For computational reasons, estimates of dates of infection were obtained separately for each of the 249 patients with E1-E2 sequences. For this, 1 sequence from each target patient, 1 from each of the 24 patients used for calibration, and 2 sequences from the PS were used in each estimate run.

Based on previous results [17], the parameters in the evolutionary and the coalescent model were given uniform or little-constrained priors (Additional file 4). The values for the parameters were saved every 500 steps and the trees every 5,000 steps. The pre-burn-in was 100,000 steps, and the burn-in was 300,000 steps. For several patients the number of steps necessary for all the parameters to have the adequate effective sample size (ESS >200) differed by an order of magnitude (from about 1 × 10^6^ to 3 × 10^7^ steps).

## Competing interests

The authors declare that they have no competing interests.

## Authors’ contributions

FG-C, MAB and AM designed research; FG-C, MAB, BW and AM performed research; FG-C and BW analyzed data; and FG-C wrote the paper. All authors read and approved the final manuscript.

## Supplementary Material

Additional file 1 Phylip-formatted file with the E1-E2 sequences used in the phylogenetic reconstruction and analyses of the outbreak. Click here for file

Additional file 2 **Nexus file corresponding to the maximum likelihood tree shown in Figure **3**.** Each sequence has been colored according to the coloring scheme used in that figure. The file can be opened and visualized with FigTree (available from http://tree.bio.ed.ac.uk/software/figtree). Click here for file

Additional file 3 **Newick file used to test the alternative hypothesis for each patient suspected to be part of the outbreak.** This tree was manually edited to replace the sequence labeled ‘test’ by the clade conformed by all the sequences derived from each patient and its likelihood was compared with that of the original tree (without the ‘test’ sequence). Click here for file

Additional file 4 **BEAST (‘Bayesian Evolutionary Analysis by Sampling Trees’) file used for deriving the date of infection of patients included in the outbreak.** This file corresponds to patient 10,067 and the sequenced used was picked at random from the cloned E1-E2 sequences available for this patient. The same procedure, using one random E1-E2 sequence, was applied to the remaining patients in the outbreak. Click here for file

Additional file 5: Figure S1 Comparison between estimates of infection dates obtained by the Bayesian method with a relaxed molecular clock implemented in BEAST (‘Bayesian Evolutionary Analysis by Sampling Trees’) and those obtained independently by the prosecution when more than one possible infection date was considered likely by the prosecution. Bayesian estimates are provided as medians and 95% highest posterior density (HPD) intervals. Prosecution estimates are shown as red (most likely date of infection as indicated during the trial) or dark-blue dots (alternative dates). Click here for file
