# Supplementary figures and images for: Molecular evolution in court: analysis of a large hepatitis C virus outbreak from an evolving source
Source: BMC Biol. 2013 Jul 19;11:76. doi: 10.1186/1741-7007-11-76 (PMC3717074; doi:10.1186/1741-7007-11-76)

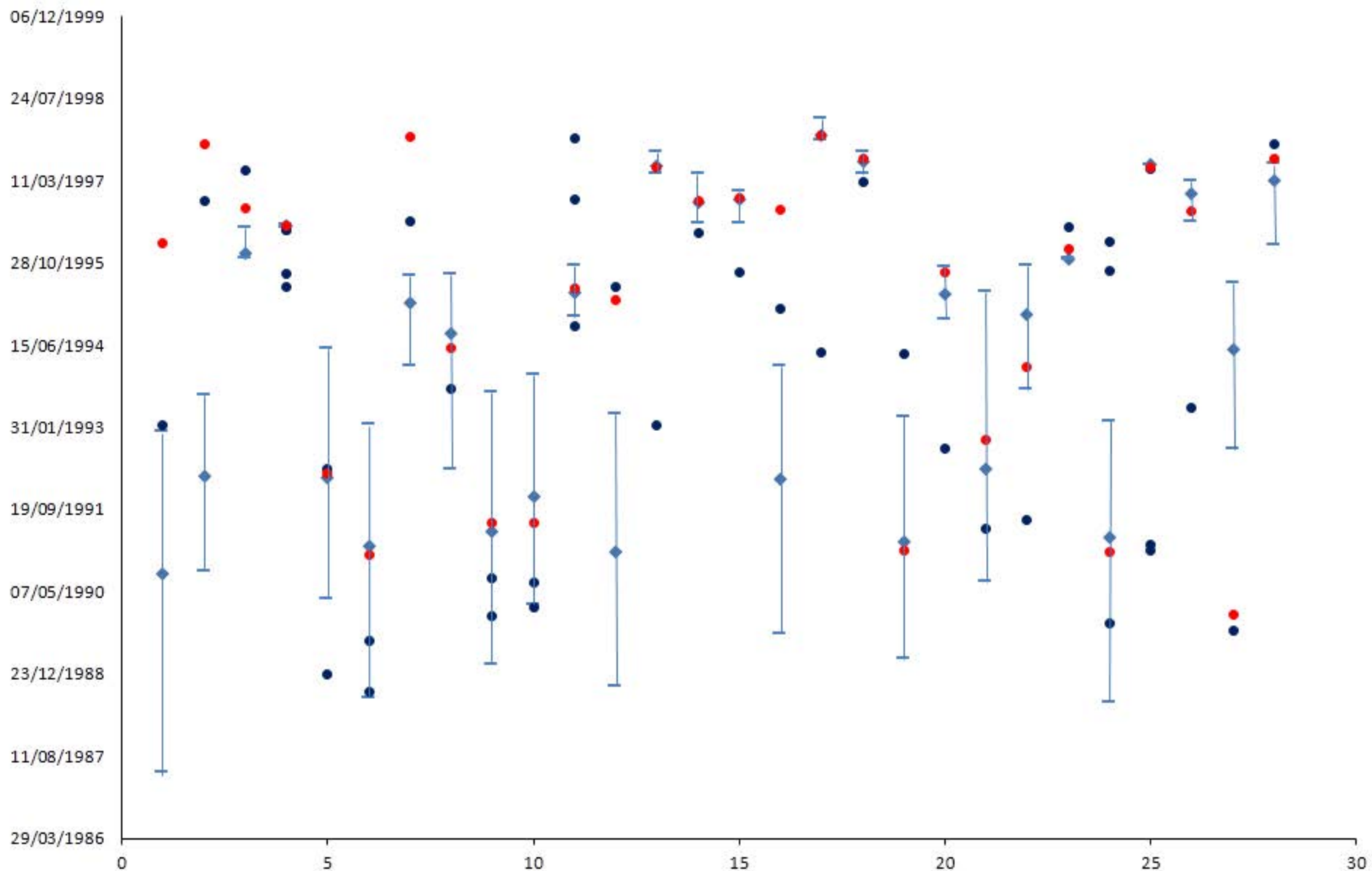

Supplement: Additional file 5: Figure S1 — Comparison between estimates of infection dates obtained by the Bayesian method with a relaxed molecular clock implemented in BEAST (‘Bayesian Evolutionary Analysis by Sampling Trees’) and those obtained independently by the prosecution when more than one possible infection date was considered likely by the prosecution. Bayesian estimates are provided as medians and 95% highest posterior density (HPD) intervals. Prosecution estimates are shown as red (most likely date of infection as indicated during the trial) or dark-blue dots (alternative dates). [file 1741-7007-11-76-S5.pdf]
